# Influence of type-I Interferon receptor expression level on the response to type-I Interferons in human pancreatic cancer cells

**DOI:** 10.1111/jcmm.12200

**Published:** 2014-01-25

**Authors:** Stephanie Booy, Casper H J van Eijck, Fadime Dogan, Peter M van Koetsveld, Leo J Hofland

**Affiliations:** a Department of Internal Medicine, Division of Endocrinology, Erasmus Medical CenterRotterdam, The Netherland; b Department of Surgery, Erasmus Medical CenterRotterdam, The Netherlands

**Keywords:** pancreatic cancer, Type-I interferons, IFNAR-1, IFNAR-2c, anti-cancer effects

## Abstract

Pancreatic cancer is a highly aggressive malignancy with limited treatment options. Type-I interferons (*e.g*. IFN-α/-β) have several anti-tumour activities. Over the past few years, clinical studies evaluating the effect of adjuvant IFN-α therapy in pancreatic cancer yielded equivocal results. Although IFN-α and-β act *via* the type-I IFN receptor, the role of the number of receptors present on tumour cells is still unknown. Therefore, this study associated, for the first time, in a large panel of pancreatic cancer cell lines the effects of IFN-α/-β with the expression of type-I IFN receptors. The anti-tumour effects of IFN-α or IFN-β on cell proliferation and apoptosis were evaluated in 11 human pancreatic cell lines. Type-I IFN receptor expression was determined on both the mRNA and protein level. After 7 days of incubation, IFN-α significantly reduced cell growth in eight cell lines by 5–67%. IFN-β inhibited cell growth statistically significant in all cell lines by 43–100%. After 3 days of treatment, IFN-β induced significantly more apoptosis than IFN-α. The cell lines variably expressed the type-I IFN receptor. The maximal inhibitory effect of IFN-α was positively correlated with the IFNAR-1 mRNA (*P* < 0.05, *r* = 0.63), IFNAR-2c mRNA (*P* < 0.05, *r* = 0.69) and protein expression (*P* < 0.05, *r* = 0.65). Human pancreatic cancer cell lines variably respond to IFN-α and-β. The expression level of the type-I IFN receptor is of predictive value for the direct anti-tumour effects of IFN-α treatment. More importantly, IFN-β induces anti-tumour effects already at much lower concentrations, is less dependent on interferon receptor expression and seems, therefore, more promising than IFN-α.

## Introduction

Pancreatic cancer is the fourth leading cause of cancer-related death in the western world [1]. Surgery is the only curative therapy, but because of early metastases and local invasion, only 15–20% of the patients are eligible for resection at time of presentation. After resection, prognosis remains poor resulting in an overall 5-year survival for patients diagnosed with pancreatic cancer of less than 5%. Several larger clinical studies have suggested the benefit of adjuvant therapy; however, no definite consensus about the optimal treatment regime has been established [2–5]. To further improve survival, research has focussed on new and other medical treatment options, like adding biological modulators as interferon (IFN) [6–7].

Interferons are known to have anti-proliferative, antiviral and immunoregulatory activities. Type-I IFNs (*e.g*. IFN-α, IFN-β and IFN-ω) are also involved in cell differentiation and anti-tumour defence processes, and besides that, they are also able to sensitize tumour cells for chemo-and radiotherapy [9–10]. Type-I IFNs act *via* the type-I IFN receptor complex, which is composed by two subunits, IFNAR-1 and IFNAR-2, of which there are three isoforms that are differently spliced from a common gene. IFNAR-2a is the soluble form and can act as a dominant negative regulator of free IFNs; IFNAR-2b is a shorter form lacking regions of the cytoplasmic domain and unable to activate JAK-STAT signalling once the receptor binds IFNs. IFNAR-2c contains the entire cytoplasmic domain and along with IFNAR-1 makes up the functional IFN receptor complex, capable of binding IFNs and inducing JAK-STAT signalling [12, 13]. Currently, IFN-α is used in the treatment of several malignancies like chronic myeloid leukaemia, metastatic melanoma, renal cell carcinoma and Kaposi sarcoma [14, 15]. Interferon-β is only used in the treatment of multiple sclerosis [16].

In experimental models *in vitro* and *in vivo*, the anti-tumour effect of IFN-α has been demonstrated [11, 17–21] and in the past years, a number of clinical studies have been conducted regarding adjuvant IFN-α therapy. The study of Picozzi *et al*. [7] reported an actuarial 5-year survival of 55%, but regrettably none of the other studies achieved an overall survival that high and also treatment toxicities were very high [6, 8].

Surprisingly, very few studies investigated the effect of IFN-β, even though some *in vitro* studies showed that IFN-β binds the receptor complex with a higher affinity and has greater anti-tumour effects than IFN-α [11, 17, 18, 22]. Although the approximate amount of receptors may determine the effect [10], the relationship of type-I IFNs receptor expression with the anti-tumour effect of IFN-α/β in pancreatic cancer cell lines is not established. Therefore, in this study, we evaluated the anti-tumour activity of IFN-α and IFN-β in 11 human pancreatic adenocarcinoma cell lines and assessed the correlation between the responsiveness to type-I IFNs and the expression of IFNAR-1 and IFNAR-2c receptors.

## Materials and methods

### Cell lines and culture conditions

The human pancreatic cell lines AsPC-1, BxPC-3, Capan-1, Capan-2, CFPAC-1, HPAF-II, Hs 700T, Hs 766T, MIA PaCa 2, PANC-1 and SU.86.86 were obtained from the American Type Culture Collection (Rockville, MD, USA). All cell lines were allelotyped and the DNA (short tandem repeat, STR) profile corresponded to the profile of the ATCC. The cells were cultured in a humidified incubator at 5% CO_2_ and 37°C. The culture medium consisted of RPMI 1640 supplemented with 5% FCS, penicillin (1 × 10^5^ U/l) and L-glutamine (2 mmol/l). Capan-1, Capan-2 and SU.86.86 were cultured in medium consisting of RPMI 1640, supplemented with 10% FCS, penicillin (1 × 10^5^ U/l) and L-glutamine (2 mmol/l). Periodically, cells were confirmed as Mycoplasm-free. Cells were harvested with trypsine (0.05%) ethylenediaminetetraacetic acid (EDTA; 0.53 mM), counted microscopically using a standard haemocytometer, resuspended in medium and plated in 24-well multiwell plates. Trypan blue staining was used to asses cell viability. Media and supplements were obtained from GIBCO Bio-cult Europe (Invitrogen, Breda, The Netherlands).

### Drugs and reagents

Human recombinant IFN-α-2b (Intron-A) was obtained from Schering-Plough Corporation (Utrecht, The Netherlands), whereas human recombinant IFN-β-1a was acquired from Serono Inc. (Rebif, Rockland, MA, USA). All compounds were stored at −20°C and stock solutions were constituted in distilled water according to the manufacturer's instruction.

### Cell proliferation assay

For each cell line, the optimal cell number plating density was determined (data not shown). After trypsinization, the cells were plated in 1 ml of medium in 24-well plates at the correct cell density. The plates were placed in a 37°C, 5% CO_2_ incubator and cells were allowed to attach overnight. The next day increasing concentrations (0–1000 IU/ml) of IFN-α or IFN-β were added. Each treatment was performed in quadruplicate. After 3 and 7 days of treatment, the cells were harvested for DNA measurement. For the 7-day experiments, the medium was refreshed after 3 days and compounds were added again. Measurement of total DNA contents was performed with the bisbenzimide fluorescent dye (Hoechst 33258; Boehring Diagnostics, La Jolla, CA, USA) as previously described [23].

### Quantitative RT-PCR

For the detection of IFN receptors (IFNAR-1, IFNAR-2 total, IFNAR-2b and IFNAR-2c) and the housekeeping gene hypoxanthine phosphoribosyltransferase (HPRT), mRNA expression was evaluated by quantitative RT-PCR in all 11 pancreatic adenocarcinoma cell lines.

The isolation of total RNA (tRNA), complementary DNA (cDNA) synthesis and the primer and probe sequences that were used for the detection of IFNAR-1, IFNAR-2 total, IFNAR-2b, IFNAR-2c and HPRT have been described previously. The soluble form of IFNAR-2a subunit was calculated indirectly by subtracting IFNAR-2b and IFNAR-2c from IFNAR-2 total [24]. All the primer and probe sequences were purchased from Biosource (Nivelles, Belgium). The primer set that was used to detect an IFN response (IFN stimulated gene 56; ISG56) was purchased from Applied Biosystems (Foster City, CA, USA) (Hs00356631).

Dilution curves were constructed for calculating the PCR efficiency for every primer set and have been described by Koetsveld *et al*. [24]. After efficiency correction of target and reference gene transcripts (HPRT), the comparative threshold method, 2^−ΔCt^ was used to calculate the relative expression of genes [25]. Interferon treatment did not change HPRT mRNA expression. As a positive control for the PCR reactions of HPRT and type-I IFN receptors, human cDNA of human carcinoid tumour cells was amplified in parallel with the cDNA samples [26].

### Protein extraction

After trypsinization, the cells were plated in 2 ml of medium in 6-well plates and placed in a 37°C, 5% CO_2_ incubator and allow to grow until a confluence of 90% was observed.

Cells were washed with ice-cold PBS. Whole-cell lysates were prepared by adding 200 μl ice-cold RIPA lysis buffer (Pierce Biotechnology Inc., Rockford, IL, USA) with the addition of 1% Halt Phosphatase Inhibitor Cocktail (Pierce Biotechnology Inc.) to each well and incubated for 1 min. on ice. Cell lysates were transferred to labelled tubes and incubated for 15 min. on ice (mixing every 5 min.) and spun down at 18,000 × *g* at 4°C. Supernatants were stored at −80°C.

With the dye-binding assay (Bio-Rad Protein Assay), the total amount of protein was calculated. Bovine serum albumin (BSA) was used as standard curve and a spectrophotometer set to 595 nm as reader.

### Western blotting

Total protein solution (50 μg) diluted in a water solution containing 25% SDS sample buffer was denatured (2–3 min. in a bath at 95°C) and separated by electrophoresis on 10% SDS-page gel. After electrophoresis, proteins were transferred onto a nitrocellulose membrane. The membranes were blocked in 0.1% Tween 20-PBS/3% non-fat dry milk for 1 hr and incubated overnight at 4°C with the primary antibody (human IFNAR1 (mouse monoclonal antibody; concentration 1:1000; Sigma-Aldrich, Zwijndrecht, The Netherlands) and human IFNAR2c (monoclonal antibody 27D11, kindly provided by Dr. E. Croze, International Review of Investigational Science, San Francisco Bay Area, USA; concentration 1:700). After three times 5 min. washing in 0.1% Tween 20-PBS, the membranes were incubated, for 1 hr at room temperature, with the secondary antibody (Alexa Fluor® goat antimouse IgG; concentration 1:15,000; Invitrogen). Starting from the incubation with the secondary antibody, membranes were kept in dark condition. After the incubation with the secondary antibody, membranes were washed twice 5 min. with 0.1% Tween 20-PBS and finally once 5 min. with only PBS.

Using the odyssey infrared imaging system (LI-COR Biosciences, Cambridge, UK), immunodetection was performed. The optical density of the sized bands was measured using the Odyssey molecular imaging software (LI-COR Biosciences). Relative expression of total IFNAR-1 or IFNAR-2c was calculated as a ratio to the expression of *beta*-actin.

### DNA fragmentation (Apoptosis)

Cells were plated, according to the optimal plating density of each cell line, in 1 ml of medium in a 24-well plate and allowed to attach overnight. The next day, medium was replaced with 1 ml fresh medium containing 1000 IU/ml IFN-α or IFN-β. Each treatment was performed in quadruplicate. After 3 days of treatment, apoptosis was measured using a commercially available ELISA kit (Cell death detection ELISA^plus^, Roche Diagnostics GmbH, Penzburg, Germany) according to the manufacturer's instructions. Apoptosis was expressed as percentage of control (untreated) cells, and data were corrected for the total DNA content in each well.

### Staining of apoptotic cells

Cells were plated and treated in the same manner and order as in the DNA fragmentation section, described above. After 3 days of treatment, cells were washed twice with PBS and fixed for 10 min. with methanol/acetic acid (3:1). After fixation, cells were washed twice with distilled water and incubated for 8 min. with 5 μg/ml Hoechst 33258 (Sigma-Aldrich). After the incubation, cells are washed again twice with distilled water and apoptotic cells were evaluated under fluorescence microscope (Axiovert 200M, HXP 120 external lamp). Cells with condensed or fragmented chromatin were considered apoptotic [27].

### Statistical analysis

All experiments were carried out at least twice, with exception of the western blot, and gave comparable results. For statistical analysis, GraphPad Prism 5.0 (GraphPad software, San Diego, CA, USA) was used. Concentrations that induced 50% growth inhibition (IC_50_) and maximal inhibitory effects were calculated using non-linear regression curve fitting program. The comparative statistical evaluation among groups was performed by a one-way anova test. When significant differences were found, a comparison between groups was made using the Newman–Keuls test. The unpaired Student's *t*-test was used to analyse differences in concentration-effect curves [IC_50_ and maximal inhibitory effect (Emax)]. After log-log transformation, the results were normally distributed and correlation analyses were performed with Pearson's coefficients. In all analyses, values of *P* < 0.05 were considered statistically significant. Data are reported as mean ± SEM.

## Results

### Anti-proliferative effect of type-I IFNs

**Figure 1:**
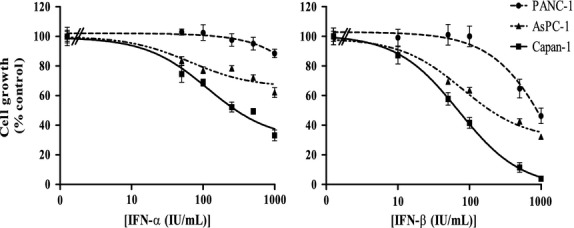
Treatment effects on cell proliferation after 7 days of incubation with increasing concentrations of interferon-α (IFN-α) and IFN-β in three human pancreatic cancer cell lines. Values are expressed as the percentage of control and represent the mean ± SEM of at least two independent experiments in quadruplicate.

After 7 days of incubation, IFN-α significantly suppressed the growth in eight of the 11 cell lines; IFN-β significantly suppressed the growth of all the cell lines (Table 1). The effects of both type-I IFNs were time-and dose-dependent. The maximal inhibition of cell proliferation induced by both compounds was significantly higher after 7 days, compared with 3 days of incubation (data not shown). Furthermore, the overall growth inhibitory effect of IFN-β after 7 days was significantly more potent than the growth inhibitory effect of IFN-α (*P* < 0.0001). Figure 1 illustrates three cell lines, AsPC-1, Capan-1 and PANC-1, which represent the spectrum of effects of IFN-α/-β treatment in the 11 cell lines. In addition, the maximal inhibitory effect of IFN-α is correlated with the maximal inhibitory effect of IFN-β (*P* < 0.05; *r* = 0.61).

**Table 1:** IC_50_ and maximal inhibitory effect (Emax) of 11 pancreatic adenocarcinoma cell lines after 7 days of incubation with IFN-α and IFN-β

Cell lines	Alpha		Beta	
	IC_50_ (IU/ml)	Emax (%)	IC_50_ (IU/ml)	Emax (%)
AsPC-1	>1000	38 ± 3.3***	272	68 ± 1.7***
BxPC-3	>1000	20 ± 4.2*	114	89 ± 2.3***
Capan-1	378	67 ± 3.6***	70	96 ± 1.2***
Capan-2	>1000	10 ± 3.6	>1000	43 ± 2.9***
CFPAC-1	841	54 ± 2.6***	131	100 ± 1.5***
HPAF-II	>1000	21 ± 4.3***	192	94 ± 2.0***
HS 700T	>1000	5 ± 4.2	272	62 ± 1.8***
Hs 766T	>1000	17 ± 3.2**	169	76 ± 1.7***
MIA PaCa2	>1000	14 ± 3.6*	422	75 ± 1.4***
PANC-1	>1000	11 ± 2.9	710	54 ± 5.3***
SU.86.86.	>1000	17 ± 2.3***	>1000	44 ± 2.4***
	378–>1000†	25 ± 3.4	70–>1000†	73 ± 2.2

* *P* < 0.05

** *P* < 0.01 and

*** *P* < 0.001 *versus* control.

The IC_50_ is expressed as the concentration needed for 50% of cell growth reduction; concentrations that exceeded 1000 IU/ml were mentioned as >1000.

† Mean IC_50_ of IFN-α and-β is expressed as the range.

The maximal inhibitory effect is expressed as the percentage inhibition compared with the untreated control ± SEM.

### Effect on apoptosis of type-I IFNs

**Figure 2:**
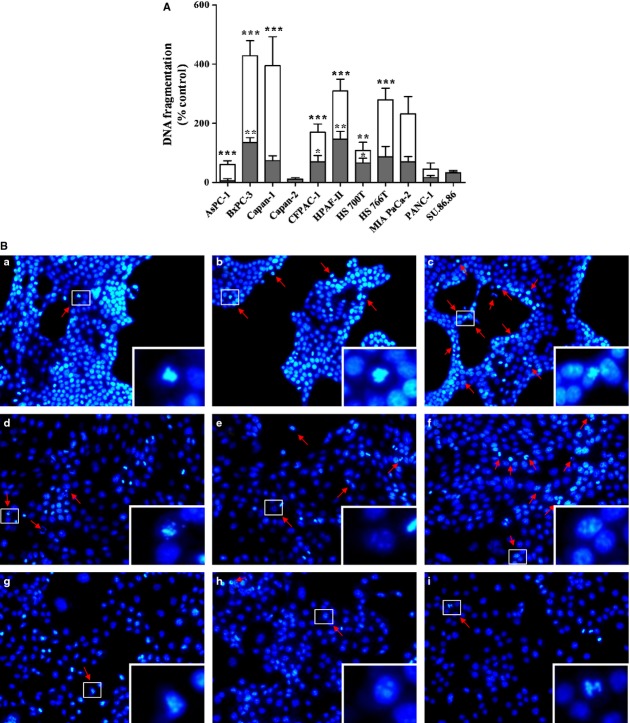
(A) Effects of interferon-α (IFN-α) (greys bars) and IFN-β (white bars) treatment on apoptosis (DNA fragmentation) in 11 human pancreatic cancer cell lines. The cells were incubated for 3 days with 1000 IU/ml of IFN-α or IFN-β. Values are absorbance units and are expressed as the percentage of change in DNA fragmentation compared with control. Data are the mean ± SEM of at least two independent experiments in quadruplicate. *P < 0.05, **P < 0.01 and ***P < 0.001 *versus* control; (B) visualization of apoptosis (nuclear condensation, fragmentation and forming of apoptotic bodies) with Hoechst 33258 after 3 days without treatment (A, D, G), IFN-α (B, E, H) or IFN-β (C, F, I) treatment in Capan-1 (A–C), CFPAC-1(D–F) and PANC-1 (G–I).The red arrows indicate condensated or fragmented nuclei or apoptotic bodies. Original magnification ×200. The inserts represent the magnification (×800) of apoptotic areas indicated by the white outline.

To evaluate the effect of type-I IFNs on apoptosis, we first measured the percentage of DNA fragmentation after 2, 4, 8, 24, 48 and 72 U of incubation with 1000 IU/ml IFN-α or 1000 IU/ml IFN-β in the relatively sensitive cell line BxPC-3 (data not shown). After 8 hrs of incubation, the effect of IFN-β on apoptosis became apparent. Starting from 24 hrs, the effect of IFN-α and IFN-β on DNA fragmentation was already significant and continued to increase significantly up to 72 hrs of IFN incubation (*P* < 0.05). As BxPC-3 is a relatively sensitive cell line and because the effect of IFN-α is less pronounced than the effect of IFN-β, we decided to measure the amount of induction of DNA fragmentation of the remaining 11 cell lines only after 72 hrs (Fig. 2A). In total, of the 11 cell lines, four cell lines showed, compared with the untreated control, a significant increase in DNA fragmentation after 1000 IU/ml of IFN-α treatment (CFPAC-1 and HS 700T; *P* < 0.05; BxPC-3 and HPAF-II; *P* < 0.01). After 3 days of IFN-β treatment (1000 IU/ml), eight of the 11 cell lines showed a significant increase in DNA fragmentation (*P* < 0.001; only HS 700T; *P* < 0.01). Only Capan-2, Panc-1 and SU.86.86 did not show a significant increase in DNA fragmentation after 3 days of IFN-α nor IFN-β treatment. In addition, the maximal effect of IFN-α on DNA fragmentation was significantly correlated with the maximal effect on DNA fragmentation of IFN-β (*P* < 0.001, *r* = 0.87). Moreover, the maximal inhibitory effect of IFN-β on proliferation was significantly correlated with the maximal effect of IFN-β on DNA fragmentation (*P* < 0.05, *r* = 0.65).

Besides the quantitative measurement of DNA fragmentation, we also visualized, in all 11 cell lines, nuclear condensation, fragmentation and apoptotic bodies with the Hoechst 33258 staining. Figure 2B shows the Hoechst staining of Capan-1 a cell line with much DNA fragmentation, the PANC-1 a cell line with very little DNA fragmentation as well as CFPAC-1, a cell line with an intermediate increase in DNA fragmentation, after IFN-α/-β treatment. The Hoechst 33258 staining of the three cell lines that is shown was in accordance with the DNA fragmentation measurement; this also applied to the remaining eight cell lines (data not shown).

### Expression of type-I IFN receptor mRNA

**Figure 3:**
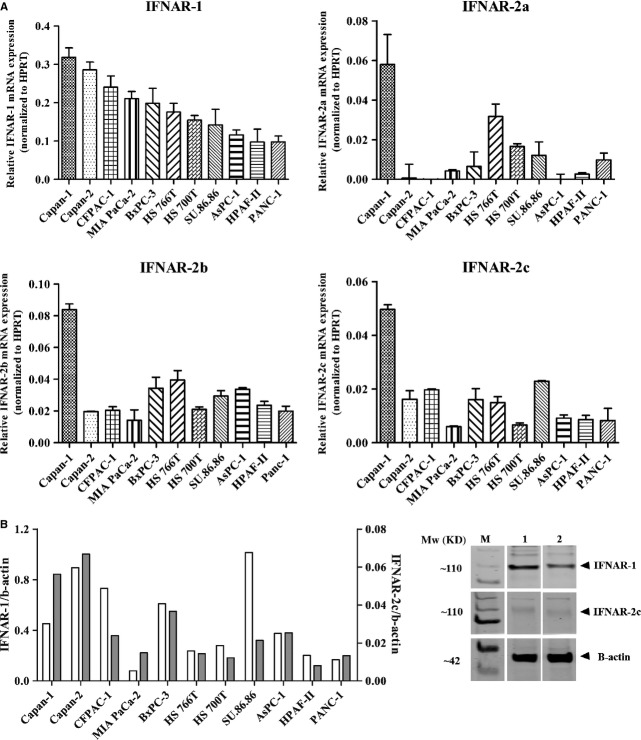
(A) Relative expression of IFNAR-1, IFNAR-2a, IFNAR-2b and IFNAR-2c mRNA in 11 human pancreatic adenocarcinoma cell lines, normalized to HPRT mRNA. Values represent mean ± SEM; (B) Right panel: Western blot analysis of high and low IFNAR-1 and IFNAR-2c protein expression in two human pancreatic cancer cell lines. Both IFNAR-1 and IFNAR-2c are expressed at ˜100 kD, previously reported to be the main Mw of both IFNAR receptors. b-actin is expressed at 42 kD (M=marker, 1. Capan-1, 2. MIA PaCa-2). Left panel; IFNAR-1 (left axis, white bars) and IFNAR-2c (right axis, grey bars) protein band density relative to b-actin band density in 11 human pancreatic adenocarcinoma cell lines.

We analysed the receptor mRNA expression of type-I IFNAR by quantitative RT-PCR in the 11 pancreatic adenocarcinoma cell lines. As shown in Figure 3A, all cell lines expressed IFNAR1 and IFNAR2, but with great variability. IFNAR-1 expression was considerably higher (on the average 3.6-fold) than IFNAR-2 total (sum of IFNAR2a, IFNAR2b and IFNAR2c isoforms) expression. The expression of the IFNAR-2 total was significantly correlated with both the expression of the IFNAR-2b (*P* < 0.0001, *r* = 0.92) and the IFNAR-2c (*P* < 0.005, *r* = 0.78). Furthermore, the expression of the IFNAR-1 mRNA is significantly correlated with the expression of the IFNAR-2c (*P* < 0.05, *r* = 0.66). Doubling time varied between cell lines (between 28.81 and 48.26 hrs). No significant correlations were found between doubling time and IFNAR1 or IFNAR2 expression (data not shown).

### Western blotting

As receptor mRNA expression does not necessarily correlate with receptor expression at the protein level, we also characterized the receptor expression at protein level by western blotting. Both the IFNAR-1 and IFNAR-2c are expressed at an anticipated [28, 29] band of ˜100 kD (Fig. 3B right panel; arrowhead), although with great variability in the level of protein expression (Fig. 3B, left panel). The expression of the IFNAR-1 was, in all of the cell lines, on average 17-fold higher than the expression of the IFNAR-2c.

The mRNA expression of the IFNAR-1 did not correlate with the expression of the IFNAR-1 at the protein level. On the other hand, the receptor expression of the IFNAR-2c subunit at mRNA level showed a significant positive correlation with the receptor expression of the IFNAR-2c at protein level (*P* < 0.05; *r* = 0.69).

### Correlation of the receptor expression with the anti-proliferative effect of type-I IFNs

Among the panel of human pancreatic cancer cell lines, we found a considerable variation in their sensitivity to type-I IFNs. Considering the fact that there was also a lot of variation in the expression of the receptor complex of IFNAR-1 and IFNAR-2c, which is responsible for initiating signal transduction, we determined the relationship between the maximal inhibitory effects of IFN-α and IFN-β and the IFNAR-1 and IFNAR-2c mRNA expression levels. Overall, only a significant correlation was found between the maximal inhibitory effect of IFN-α and the expression of IFNAR-2c (*P* < 0.05, *r* = 0.63).

However, after evaluation of the individual cell lines, there was one cell line that responded completely different to type-I IFNs compared with the other cell lines. For this cell line, *e.g*. Capan-2, it is remarkable that there is a poor response to IFN-α and IFN-β, since this cell line does express a significant number of IFNAR-1 and IFNAR-2c receptors to initiate an effect. This could be due to a defect in post-receptor signal transduction, which was illustrated by the fact that there was only a very low up-regulation of ISG56 after IFN-α (4.47 ± 0.45-fold compared with control) and IFN-β (7.00 ± 0.50-fold compared with control) treatment. This was not in line with the amount of up-regulation of ISG56 in the BxPC-3 cell line after IFN-α (41.24 ± 12.1-fold) or IFN-β treatment (99.47 ± 5.61-fold), a cell line with nearly the same amount of IFNAR expressed.

**Figure 4:**
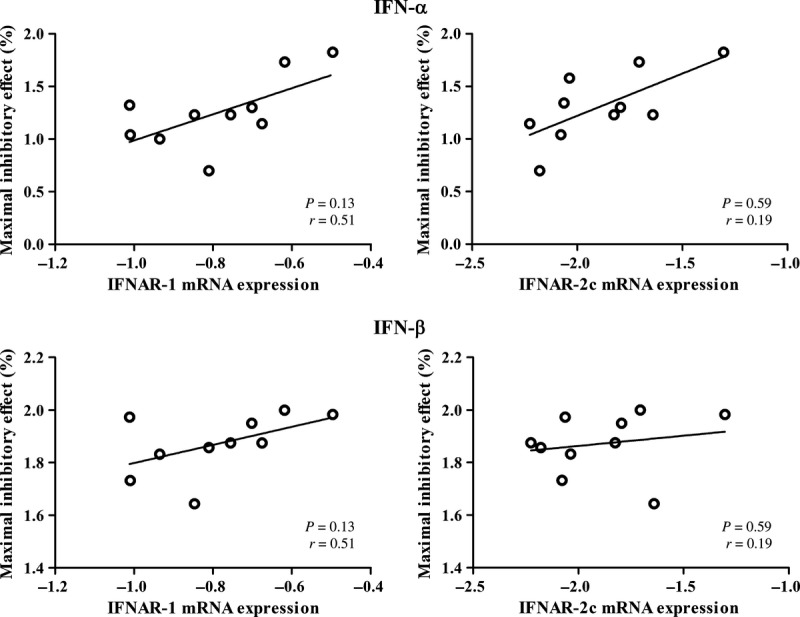
Correlation of the maximal inhibitory effect of interferon-α (IFN-α; 1000 IU/ml) and IFN-β (1000 IU/ml) with the expression level of IFNAR-1 and IFNAR-2c mRNA in 10 human pancreatic adenocarcinoma cell lines (log-log transformed scale). The maximal inhibitory effect is expressed as percentage inhibition compared with untreated control. There was a significant correlation between the expression of IFNAR-1 mRNA and the maximal inhibitory effect of IFN-α (*P* < 0.05, *r* = 0.63). A significant correlation was found as well between the expression of the IFNAR-2c and the maximal inhibitory effect of IFN-α (*P* < 0.05, *r* = 0.69). However, there was no significant correlation regarding the maximal inhibitory effect of IFN-β and the expression of the IFNAR-1 or IFNAR-2c mRNA.

**Figure 5:**
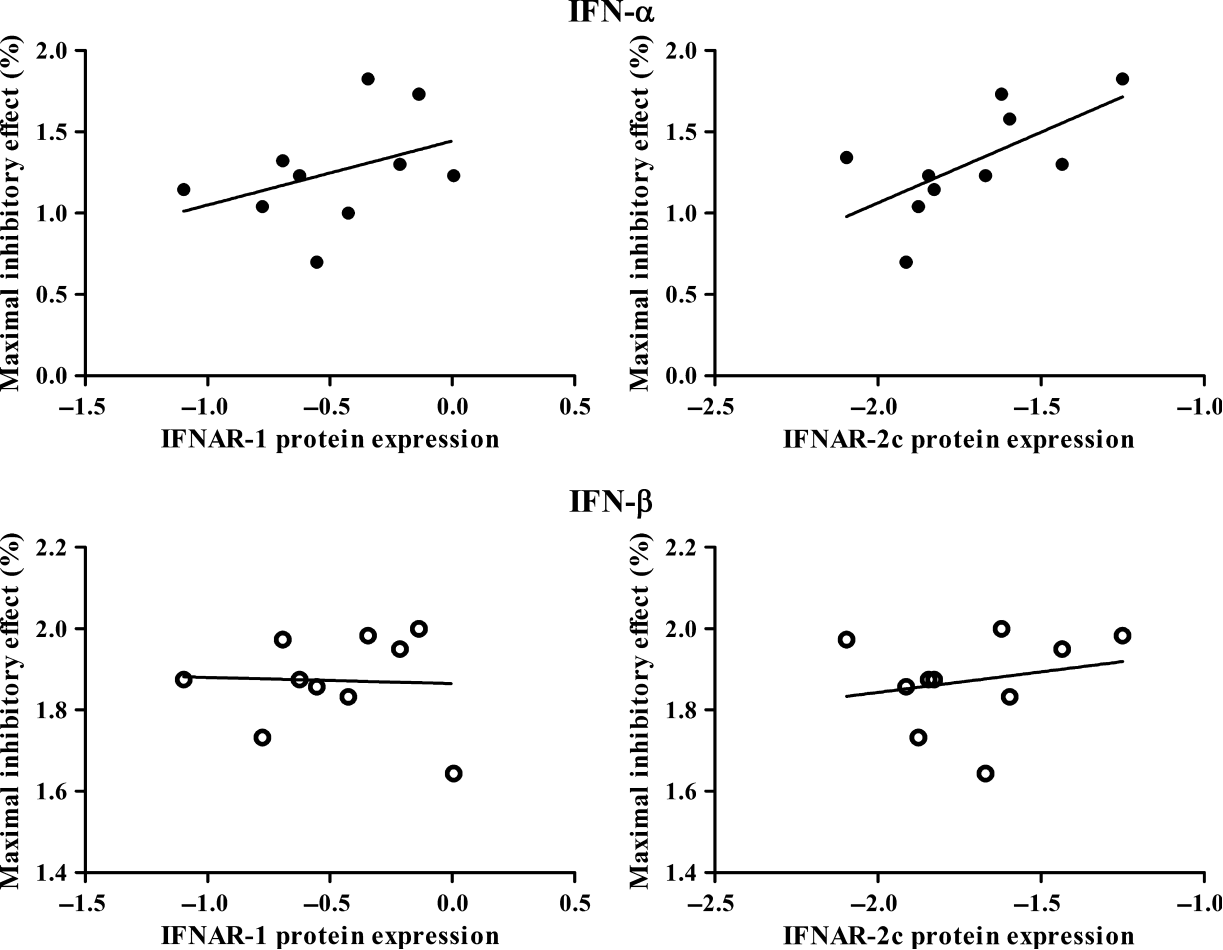
Correlation of the maximal inhibitory effect of interferon-α (IFN-α; 1000 IU/ml) and IFN-β (1000 IU/ml) with the expression of IFNAR-1 and IFNAR-2c at protein level in 10 human pancreatic adenocarcinoma cell lines (log-log transformed scale). The maximal inhibitory effect is expressed as percentage inhibition compared with untreated control. There was a significant correlation between the expression of IFNAR-2c protein and the maximal inhibitory effect of IFN-α (*P* < 0.05, *r* = 0.65); this correlation was not found for IFN-β. No correlation was found between the IFNAR-1 mRNA expression and the maximal inhibitory effect of IFN-α or IFN-β.

For this reason, we excluded Capan-2 from further correlation analysis. Figures 4 and 5 show the correlations between the growth inhibitory effect of IFN-α and IFN-β and IFNAR mRNA and protein expression, respectively, in the remaining 10 cell lines. There was a significant correlation between the IFNAR-1 mRNA expression and the response to IFN-α (*P* < 0.05, *r* = 0.63; Fig. 4, left upper panel). This correlation was also found regarding IFNAR-2c mRNA expression and the response to IFN-α (*P* < 0.05, *r* = 0.69; Fig. 4, right upper panel). Furthermore, there was a significant correlation between the IFNAR-2c mRNA expression and the IFNAR-2c protein expression (*P* < 0.01, *r* = 0.77). The IFNAR-2c protein expression also correlated significantly with the response to IFN-α (*P* < 0.05, *r* = 0.65; Fig. 5, right upper panel). IFNAR2a and IFNAR2b expression did not correlate with the effects of IFN-α or IFN-β treatment.

## Discussion

Pancreatic cancer is a highly aggressive malignancy, with very limited treatment outcome. The effect of adjuvant treatment modalities like chemo-and radiotherapy is still marginal. To improve survival in patients with pancreatic cancer, additional treatment options are clearly required [2, 3]. Several years ago, a number of clinical studies have been conducted regarding adjuvant IFN-α therapy in the treatment of pancreatic cancer. Some studies reported a remarkable increase in the 2-and 5-year survival [6, 7, 30]. On the other hand, the only randomized clinical trial did not show a significant increase in overall survival, although the increased median survival implicated that some patients in the experimental arm benefited from adjuvant IFN-α therapy [31]. Despite that some *in vivo* and *in vitro* studies have investigated the role of type-I IFNs, in particular IFN-α in pancreatic cancer, the importance of the IFN receptor expression in relation to the effect of type-I IFNs is not clarified. Previous research of Vitale *et al*. [17] and Saidi *et al*. [20, 32, 33] established a trend towards the importance of the IFN receptor in the response of IFN-α and –β; however, the number of cell lines used in these studies was very low. Furthermore, although some studies did show promising results [11, 17, 18, 22], the effect of IFN-β in the treatment of pancreatic cancer still remains underexposed. Therefore, in this study, we evaluated the type-I IFN receptor expression in a large panel of available human pancreatic cancer cell lines and associated the receptor expression with the anti-tumour potencies of IFN-α and IFN-β.

In the panel of 11 human pancreatic adenocarcinoma cell lines, there was a considerable variability in the response to the type-I IFNs. Overall, IFN-β is a significantly more potent inhibitor of cell proliferation compared with IFN-α. IFN-β inhibited cell proliferation already at very low concentrations (10–50 IU/ml) in the majority of the cell lines, which is in agreement with the study of Vitale *et al*. [17] and Jost *et al*. [18]. The maximal inhibitory effect of IFN-α significantly correlated with the maximal inhibitory effect of IFN-β. Consequently, cell lines that achieve a higher maximal inhibitory effect with IFN-α will also achieve a higher maximal inhibitory effect with IFN-β.

Type-I IFNs are also known to induce apoptosis, which can act *via* the intrinsic mitochondria–mediated pathway or the extrinsic death receptor–induced pathway, both resulting in nuclear condensation and fragmentation, followed by fragmentation of the cell into apoptotic bodies. Both type-I IFNs were able to induce apoptosis; however, the increase in DNA fragmentation after IFN-β therapy was considerably more potent compared with the increase in DNA fragmentation after IFN-α treatment. The increase in DNA fragmentation was also visualized by Hoechst 33258 and was consistent with the quantitative DNA fragmentation measurements.

By quantitative RT-PCR and western blotting, we demonstrated that all pancreatic cell lines expressed the IFNAR-1 and IFNAR-2c receptors, although receptor expression levels varied considerably. The maximal inhibitory effect of IFN-α was positively correlated with the expression of IFNAR-2c. Remarkably, one cell line, Capan-2, did express a significant amount of the type-I IFN receptor, but showed only a marginal response to IFN-α and-β. Type-I IFNs act *via* several signalling pathways [34]. Like in many different types of cancers, there can be a defect in the IFN post-receptor signalling pathway, resulting in the absence of an up-regulation of IFN-stimulated genes. For this reason, we measured the up-regulation of ISG56 after IFN-α and IFN-β treatment in the Capan-2 cell line. Compared with BxPC-3, Capan-2 showed only very low ISG56 up-regulation. Therefore, a defect in the post-receptor signalling pathway in Capan-2 is likely. The development of pancreatic cancer often occurs through the accumulation of genetic mutations, including frequent mutations in the KRAS (Kirsten rat sarcoma viral oncogene homolog), p53, CDKN2A (p16) and SMAD4 (DPC4) [35]. The presence or absence of genetic mutations in these cell lines could determine the amount of IFN receptors expressed and/or influence the effect of IFN-α and-β. The cell lines included in our study displayed a wide variability in genetic alterations. There was no difference between wild-type and mutated cell lines in their response to IFN-α and-β treatment, nor in the number of type-I IFNARs. Nevertheless, it is notable that Capan-2 is the only wild-type p53 cell line in this panel of pancreatic cancer cell lines. Literature data shows that type-I IFNs can up-regulate p53, which can initiate apoptotic pathways [36]. The fact that we did observe an up-regulation of apoptosis in the mutated p53 cell lines, while very little induction of apoptosis in the Capan-2 wild-type p53 cell line, strengthens the concept that the Capan-2 cell line has an impaired post-receptor IFN signalling pathway. For this reason, the Capan-2 cell line was excluded from the further analysis. In the remaining 10 cell lines, there was a significant positive correlation between the IFNAR-1 mRNA, but not protein, expression and the maximal inhibitory response to IFN-α. In addition, IFNAR-2c mRNA and protein expression showed a positive correlation with the maximal inhibitory effect of IFN-α. Our findings in human pancreatic cancer cells endogenously expressing the IFNAR2c add to the observations by Wagner *et al*. [10], who showed in melanoma, breast cancer and lung fibrosarcoma cell models that transfection and overexpression of IFNAR-2c enhances their sensitivity for type-I IFNs *in vitro* and *in vivo*. Based on these findings, it is concluded that the number of IFNAR-2c receptors can be of predictive value in determining responsiveness of human pancreatic cancer cells to IFN-α therapy. However, one should realize that in selected pancreatic cancers, reflecting our observations in Capan-2 cells, defects in IFNAR signalling can occur, rendering such cancers insensitive to IFN treatment.

Although IFN-α and-β act *via* the same receptor complex, both cytokines display functional differences. In addition to the fact that IFN-α and-β share only 35% of their sequence identity and that IFN-β, unlike IFN-α, is a glycosylated protein, a study by Johns *et al*. demonstrated that IFN-β has a 10-fold higher binding affinity with the receptor complex compared with IFN-α [37]. Moreover, IFN-β induced an up-regulation of 338 genes in human fibrosarcoma cells, whereas IFN-α induces an up-regulation of only 130 genes [38]. A different interaction with the type-I IFN receptor complex between IFN-α and-β can be an explanation of the more potent activity of IFN-β as well. Recently, an elegant study by de Weerd *et al.,* using IFNAR1 and IFNAR2 knockout mice, demonstrated that IFN-β can induce functional signal transduction, *via* the IFNAR-1, independently of the IFNAR-2c receptor [39]. In addition, it was found that 104 unique genes were induced by this IFNAR1-IFN-β signalling axis. These observations may form an explanation of the lack of correlation between the IFNAR-1 and/or IFNAR-2c receptors and the maximal inhibitory effect of IFN-β, as we found in our study, but also for the much higher potency of the anti-tumour effects of IFN-β, compared with IFN-α.

Although hypothetical, several potential pathways may be involved in the above indicated differential direct anti-tumour activities of IFN-α and IFN-ß. First, IFN-β may be more potent than IFN-α in stimulating the protein kinase dependent on double-stranded RNA (PKR). PKR is involved in the regulation of protein synthesis and the action of transcription factors and is thereby able to control several cellular processes, including cell growth [40, 41]. Secondly, IFN-β may be more effective in down-regulating cdk activity or up-regulating cdk-inhibitory proteins, as the compound was shown to be more potent than IFN-α in inducing cell cycle arrest in human pancreatic cancer cell lines [17]. Thirdly, fewer or less effective survival mechanisms may be induced (*i.e*. up-regulation of the EGFR, stimulation of STAT-3, induction of SOCS-1 and-3 or the stimulation of the MAPK cascade; [41]) after IFN-β treatment compared with IFN-α treatment. However, to the best of our knowledge, there are no studies that made a direct comparison between the effects of IFN-α and IFN-ß on PKR, cdk's and survival pathways in human pancreatic cancer cells.

Regarding IFN-β, the concentration required to reduce cell growth with 50% ranged from 70 to over a 1000 IU/ml. These concentrations seem not easily reached in serum of human healthy volunteers after s.c. administration (4–10 IU/ml after four doses of 18 MIU IFN-β at 48-hr intervals) [42]. However, the recently developed PEGylated form of IFN-β, which is currently being tested in phase III clinical trial (ADVANCE) in patients with multiple sclerosis seems very promising in this respect. In experimental models, after a single s.c. dose of PEG-IFN-β (3.0 MIU/kg in monkeys), serum concentrations of 100 IU/ml were reached after 20 hrs [43, 44]. Although this serum concentration is for some cell lines still not high enough, it does get into the sensitivity range to IFN-β of several well responding pancreatic cancer cell lines.

Our study showed four cell lines in which IFN-α and-β had only a marginal anti-tumour effect, but over time, an increasing trend in anti-tumour activity was observed. This advocates for a longer treatment period, combined with DNA damaging agents like 5-fluorouacil, gemcitabine or radiotherapy. Tomimaru *et al*. confirms this hypothesis and showed that IFN-β combined with gemcitabine was able to induce synergistically anti-tumour effects even in the pancreatic cancer cells with low IFN receptor expression [22]. Taken this together, this seems promising and further *in vivo* research is definitely necessary. Furthermore, the interaction of type-I IFNs with the cells representing the host immune response cannot be neglected as the relevance of cancer immunoediting is becoming clearer [45].

In conclusion, IFN-β is a significant more potent growth inhibitor in pancreatic cancer than IFN-α. Although there is a lot of heterogeneity among the panel of pancreatic cancer cell lines, there is a significant correlation between the expression level of the IFNAR-2c receptor and the maximal inhibitory effects of IFN-α. This study provides for the first time extensive and well-substantiated evidence that the expression of these receptors in pancreatic cancer can be of predictive value in the responsiveness to the direct tumour growth inhibitory effects IFN-α therapy. More importantly, IFN-β induces a tumour growth inhibitory effect already at lower concentrations, is less dependent on receptor status and seems, therefore, more promising than IFN-α.
